# Future pharmacy practitioners’ insights towards integration of artificial intelligence in healthcare education: Preliminary findings from Karachi, Pakistan

**DOI:** 10.1371/journal.pone.0314045

**Published:** 2025-02-12

**Authors:** Tahmina Maqbool, Humera Ishaq, Sadia Shakeel, Ayeshah Zaib un Nisa, Hina Rehman, Shadab Kashif, Halima Sadia, Safila Naveed, Nazish Mumtaz, Sidra Siddiqui, Shazia Jamshed

**Affiliations:** 1 Faculty of Pharmacy, Department of Pharmaceutics, Hamdard University, Madinat al-Hikmah, Karachi, Pakistan; 2 Faculty of Health and Medical Sciences, Hamdard University, Karachi, Pakistan; 3 Faculty of Pharmaceutical Sciences, Department of Pharmacy Practice, Dow College of Pharmacy, Dow University of Health Sciences, Karachi, Pakistan; 4 Faculty of Pharmacy, Department of Pharmacy Practice, Hamdard University, Madinat al-Hikmah, Karachi, Pakistan; 5 Department of Pharmacy Practice, Institute of Pharmaceutical Sciences, Jinnah Sindh Medical University, Karachi, Pakistan; 6 Faculty of Pharmacy, Department of Pharmacy Practice, Salim Habib University, Karachi, Pakistan; 7 Faculty of Pharmacy, Department of Pharmacy Practice, Jinnah University for Women, Karachi, Pakistan; 8 Faculty of Pharmacy, Department of Pharmaceutical Chemistry, University of Karachi, Karachi, Pakistan; 9 Faculty of Pharmacy, Benazir Bhutto Shaheed University Lyari, Karachi, Pakistan; 10 Department of Pharmacy Practice, School of Pharmacy, International Medical University, Kuala Lumpur, Malaysia; Jouf University, SAUDI ARABIA

## Abstract

In an evolutionary era of medical education, “Artificial intelligence” (AI) is applied to replicate human intellect, encompassing abilities, logical reasoning and effective problem-solving skills. Previous research has explored the attitude of medical and dental students, toward the assimilation of AI in medicine; however, a significant gap exists in appraising the understanding and concerns of pharmacy students. Therefore, the current study was designed to explore undergraduate pharmacy students’ perceptions of integrating AI into education and practice. **Methods**: A cross-sectional study was conducted among final-year pharmacy students from different public and private sector universities in Karachi. The sample size on 60% anticipated response rate and 99% CI was calculated to be 390. Data was collected after acquiring ethical approval using convenient sampling. Frequency and percentage of the socio-demographic features were analyzed and then goodness of fit and Pearson’s chi-squared test of correlation was applied. Results were considered significant when p < 0.05. **Results**: The overall response rate of the study was 67%. More than 80% of the respondents were female. The students 35% (n = 202) strongly agreed and 59% (n = 334) agreed that AI plays an important role in healthcare, (χ2 = 505.6, p < 0.001). Around 79% (n = 453, χ2 = 384.3, p < 0.001) of students agreed on the replacement of patient care specialties with AI in the future, whereas 495 students (87%, χ2 = 682.3, p < 0.001) stated that they possess a strong comprehension of the fundamental principles governing the operation of AI. More than 80% of the students were comfortable in using AI terminologies (n = 475, χ2 = 598, p < 0.001) and 93% (n = 529, χ2 = 290, p < 0.001) were sure that AI inclusion in pharmacy education will develop a positive influence into the pharmacy curriculum (95%, n = 549, χ2 = 566.9, p < 0.001). A high and positive correlation was observed between the perception and willingness of students to adopt the AI changes in teaching undergraduate students (ρ = 0.491, p < 0.001). Furthermore, the outcomes showed students at private-sector universities stood out in computer literacy compared to public-sector universities (χ2 = 6.546, p < 0.05). **Conclusion:** The current outcomes revealed the higher willingness of pharmacy students towards AI-infused learning. They understood the prerequisite of having both formal and informal learning experiences on the clinical application, technological constraints, and ethical considerations of the AI tools to be successful in this endeavor. The policymakers must take action to ensure that future pharmacists have a strong foundation of AI literacy and take initiatives to foster the interests and abilities of imminent pharmacists who will spearhead innovation in the field.

## Introduction

In the evolutionary era of medical training, the incorporation of state-of-the-art technologies has become crucial in equipping prospective healthcare practitioners with the demands of a continuously advancing healthcare framework. In recent years a technology that has gained maximum traction is “Artificial Intelligence (AI)” [1]. AI involves the development of machines that can replicate human intelligence, encompassing abilities such as comprehension, logical reasoning, and effective problem-solving. AI involves the interpretation of external data, assimilating knowledge from it, and applying this acquired knowledge to accomplish defined objectives and tasks [2]. AI systems are proficient in executing a wide range of tasks to assist healthcare professionals across diverse medical domains. These functionalities encompass aiding in drug development, diagnosing diseases, monitoring health, managing medical data, implementing personalized medicine, and treatments, analyzing health plans, and surgical procedures [3].

AI encompasses various subfields like “Machine Learning” (ML), which contribute intelligence to applications either individually or collaboratively. ML involves algorithms enabling computer programs to autonomously enhance performance through experience, categorized into “supervised”, “unsupervised”, and “Reinforcement Learning (RL)” [1]. “Supervised learning” utilizes labeled information, such as interpreted X-ray images, for tasks like detecting new growths in images. “Unsupervised learning” extracts facts from unlabeled data, categorizing patients by identifying causes that match similar symptoms. “RL” involves computational activity along with (trial and error” or “expert demonstration”, developing strategies to maximize rewards—a key driver behind recent AI breakthroughs. “Deep Learning (DL)”, a subset of ML, employs multi-layered algorithms, emerging as the predominant AI method, particularly advancing image and speech recognition [4].

In radiological examination, a subset of AI has demonstrated comparable performance to doctors when it comes to lung cancer screening and risk assessment using CT scans. Additionally, it accurately measures heart shape, size and performance based on echocardiograms [5]. In pathology, deep learning algorithms replace a team of clinicians in detecting malignant metastases. Amid the COVID-19 pandemic, AI excels in interpreting chest CT imaging, offering swift and precise diagnoses, even outperforming radiologists[6,7]. Various reviews underscore AI’s potential to revolutionize medical disciplines like oncology, radiology, and pathology while acknowledging the associated tasks [8].

The development goals released by the International Pharmaceutical Federation (FIP) [9], are an essential tool for revolutionizing the pharmacy profession on a national, regional, and worldwide scale during the next ten years. FIP Development Goal 21 (Sustainability in Pharmacy) requires the pharmacy profession to implement policies, procedures, and tactics that make use of the pharmaceutical workforce to improve pharmacy and services [9]. The pharmacy profession must embrace the digital transformation that has been rapidly changing healthcare to ensure the sustainability of pharmacy practice and to provide health for all. It is expected that major tasks like referrals, patient screenings, and clinical recommendations will become automated due to “AI-assisted systems”. The role of pharmacists is poised for significant transformation, as AI becomes increasingly integrated into healthcare [10]. AI can accelerate drug identification and development by employing “machine learning models” to analyze “extensive molecular data” and identify prognostic targets [11]. Efficient analysis and sorting of clinical data are major AI potentials that may enhance access to crucial patient data [12]. AI-assisted “pharmacovigilance” can encompass a large data bank and can help scan potential adverse effects, their early detection and avoidance of risks [13]. Moreover, the implementation of AI-powered decision support systems helps in drug selection and dosage adjustment, ultimately improving patient safety by improving treatment [14].

Various organizations, including the “Association of American Medical Colleges” (AAMC) and the “Royal College of Physicians and Surgeons of Canada”, proposed that healthcare professionals undergo training in AI, data security, “AI ethics”, and the “critical evaluation of AI applications in healthcare” [15]. Assessing public attitudes and knowledge among healthcare students proves to be an effective strategy in identifying educational needs for AI curriculum decision-makers. As the integration of AI in healthcare gains momentum, the necessity for enhanced knowledge and AI-focused training for physicians and medical students becomes increasingly evident [16].

While previous research has explored the attitudes of undergraduate medical and dental students, towards AI in medicine, there is a substantial gap in understanding the perspectives and relations of pharmacy students worldwide regarding the inclusion of AI into pharmacy education and the profession [17,18]. The studies have been conducted internationally to explore the viewpoints of pharmacy students on the inclusion of AI into pharmacy practice and education; however, no such data has been reported from Pakistan[19]. Hence, the current research attempted to focus on examining the pharmacy students’ views on AI systems in pharmacy education and their willingness to integrate these systems into their learning. Therefore, keeping the paucity of data in higher education institutes in Pakistan, the current research is pertinent to exploring the knowledge of pharmacy students about AI use in pharmacy education.

## Methods

### Study design and setting

A cross-sectional study was performed among final professional pharmacy students of all public and private sector universities offering pharmacy education in Karachi. Those students who were not in the final year of the pharmacy graduate program were excluded from the study. A total of 852 students were approached for the study. The study population was extracted from three public sector universities in which PubU 1 had 56, PubU 2 had 100 and PubU 3 had 140 final year students whereas out of four private universities, PriU 1 had 100, PriU 2 had 200, PriU 3 had 16 and PriU 4 had 200 final year students.

### Sample size calculation

The sample size was calculated using the formula “n = [DEFF*Np (1-p)]/ [(d2/Z 2 1-α/2 *(N-1)+p*(1-p)]”, where the population size was kept 1000 and the anticipated response rate was 60%. At 99% CI sample size was calculated to be 390 [20].

### Survey questionnaire

The questionnaire was adopted from the recently published article on AI after obtaining prior permission to use the questionnaire [21]. Written approval was sought before conducting the study. As the questionnaire was validated and verified previously, therefore, the questionnaire was implemented after ethical approval via online Google forms. The questionnaire was divided into 3 portions. The first portion comprised of demographics; the second part comprised of students’ perceptions towards AI and the third section was about the influence of AI on pharmacy education.

### Ethical approval

The study received approval from “Hamdard University Ethical Review Committee (ERC)” via letter number ERC-FOP-2024-003. This research was conducted under the guidelines of the “international conference of Harmonization (ICH)”, and the Declaration of Helsinki” for human subjects. A section on consent was added to the online questionnaire. The form proceeded only when participants clicked the “I give consent” button.

### Data collection

After approval from the ethical committee, the recruitment of respondents was conducted. Students were contacted on their WhatsApp groups and their teachers asked them to fill out the Google form. Due to less response, the link was reshared. Data was collected from 16 April 2024 for 15 days after which data was subjected to statistical analysis.

### Statistical analysis

By using SPSS-20 statistical analysis was performed. Information in the form of data is presented in frequency (percentage). The goodness of fit test for the variables was applied. A correlation coefficient matrix was established for questions where r values and p values are highlighted. Pearson’s chi-squared test of correlation was applied using a dichotomous variable of types of universities and was compared with different questions of the questionnaire. Results were concluded significant when “p < 0.05”.

## Results

### The response rate of the study

Out of 852 final-year students in different universities in Karachi, 570 students responded to the study and completed their questionnaires giving 67% of the response rate.

### Descriptive statistics

Table 1 shows demographical details of the study population where all the participants (100%) belonged to the 22–24 years age slot with 81% (n = 459, χ^2^ = 213, p < 0.001) female respondents. 41% of students (n = 232, χ^2^ = 19.7, p < 0.001) belonged to public sector universities and 59% (n = 338) belonged to private sector universities.

**Table 1:** Demographic details of respondents.

Variables		Frequency (Percentage)	Χ^2^
Gender	Male	111 (19%)	213**
	Female	459 (81%)	
Computer literacy	Literate	234 (41%)	106.3**
	Competent	261 (46%)	
	Proficient	75 (13%)	
Computer usage for learning purposes	Never	3 (0.5%)	391.02**
	Sometimes	179 (31%)	
	Always	388 (68%)	
University	Public sector	232 (41%)	19.7**
	Private sector	338 (59%)	

** = p < 0.001. 1. “AI system will have a positive impact on pharmacy education”; 2. “Incorporating the AI system in pharmacy education will ease the learning process”; 3. “Use of AI system will prepare you for real clinical practice”; 4. “The use of the AI system in pharmacy practice would replace your future role as a pharmacist”; Willingness. “I am willing to use AI in pharmacy education systems”.

Regarding computer literacy only 13% (n = 75, χ^2^ = 106, p < 0.001) were proficient whereas most of them i.e. 46% (n = 261) were competent. 68% of students (n = 388, χ^2^ = 391, p < 0.001) always used computers for learning purposes, and only 3 students (0.5%) never used computers for study purposes.

### Perceptions of participants about AI

Table 2 depicts the awareness and insight of the students about AI. It was found that the students were aware of the AI technology (n = 453, 79%) and its concepts (n = 495, 87%). They were well-versed of the significance of AI in education (n = 472, 83%) and practice (n = 536, 94%). Students were comfortable with using AI terminologies (n = 475, 83%) and wanted to receive advanced training to improve their skills for clinical practices (n = 522, 92%).

**Table 2:** Perception of pharmacy students towards artificial intelligence (n = 570).

Perception of University student regarding AI					
Variable	“Strongly agree”	“Agree”	“Disagree”	“Strongly disagree”	Χ^2^
1. Statement 1	202 (35%)	334 (59%)	30 (5%)	4 (1%)	505.6**
2. Statement 2	122 (21%)	331 (58%)	107 (19%)	10 (2%)	384.31**
3. Statement 3	88 (15%)	407 (71%)	71 (12%)	4 (1%)	682.3**
4. Statement 4	86 (15%)	389 (68%)	88 (15%)	7 (1%)	598.5**
5. Statement 5	88 (15%)	383 (67%)	95 (17%)	4 (1%)	577.2**
6. Statement 6	177 (31%)	355 (62%)	36 (6%)	2 (0.35%)	543.4**
7. Statement 7	209 (37%)	313 (55%)	41 (7)	7 (1%)	436.2**
8. Statement 8	117 (21%)	358 (63%)	91 (16%)	4 (1%)	483.7**
9. Statement 9	104 (18%)	369 (65%)	89 (16%)	8 (1%)	517.5**
10. Statement 10	105 (18%)	374 (66%)	82 (14%)	9 (1.5%)	536.7**

** = p < 0.001; 1. “Artificial intelligence (AI) will play an important role in healthcare”; 2. “AI will replace some specialities in healthcare during my lifetime”; 3. “I understand basic AI principles”; 4. “I am comfortable with AI terminologies”; 5. “I understand AI limitations”; 6. “AI teaching will benefit my career”; 7. “All pharmacy students should receive AI teaching”; 8. “I will be confident in using AI tools at the end of my pharmacy degree”; 9. “I will have a better understanding of methods used to assess healthcare AI performance at the end of my pharmacy degree”; 10. “I will possess the knowledge needed to work with AI in routine clinical practice at the end of my pharmacy degree”.

### Student’s viewpoint on the influence of “Artificial Intelligence” (AI) on pharmacy education and their desire for its utilization

Table 3 shows the influence of AI on pharmacy education in Pakistan as viewed by the pharmacy undergraduates and their preparedness and desire to use it. The addition of AI in the pharmacy curriculum will be beneficial for future pharmacists according to 95% of students (n = 549) and it will be a positive influence on pharmacy education (n = 529, 93%) and will prepare the students for real-life clinical scenarios (n = 448, 78.6%).

**Table 3:** Influence of “Artificial Intelligence” on pharmacy education and desire to use it.

“Impact of AI on pharmacy according to students”					
Impact of AI in Pharmacy	“Strongly Agree”	“Agree”	“Disagree”	“Strongly disagree”	χ^2^
1. Statement 1	160 (28%)	369 (65%)	41 (7%)	0	290.2**
2. Statement 2	186 (33%)	356 (62%)	41 (7%)	2 (0.35%)	566.9**
3. Statement 3	119 (21%)	329 (58%)	115 (20%)	7 (1%)	382.1**
4. Statement 4	104 (18%)	220 (38%)	220 (38%)	26 (4%)	189.9**
	**“Strongly willing”**	**“Willing”**	**“Not willing”**	**“Strongly not willing”**	
5. Willingness	151 (26%)	373 (65%)	41 (7%)	4 (1%)	581.3**

** = p < 0.001. 1. “AI system will have a positive impact on pharmacy education”; 2. “Incorporating the AI system in pharmacy education will ease the learning process”; 3. “Use of AI system will prepare you for real clinical practice”; 4. “The use of the AI system in pharmacy practice would replace your future role as a pharmacist”; Willingness. “I am willing to use AI in pharmacy education systems”.

There was a mix kind of reaction among the students about AI replacing the roles of pharmacists in the future where 57% (n = 324) agreed with the concept whereas 43% (n = 246) disagreed. Willingness to use AI among the students of the pharmacy profession is very high (n = 524, 92%).

A correlation analysis between questions of students’ perception and impact regarding AI showed a very significant correlation among all questions but an exceptionally high and positive correlation was observed between the benefit of AI teaching for carriers with the question regarding AI will have a positive impact on pharmacy education (r = 0.491, p < 0.001); AI incorporation will ease the teaching and learning of pharmacy (r = 0.434, p < 0.001) and willingness of the students to learn AI (r = 0.430, p < 0.001) (Table 4).

**Table 4:** Correlation matrix of student’s perception of AI and impact of AI in pharmacy education and willingness to use it.

Perception/Impact	Statement 1	Statement 2	Statement 3	Statement 4	Willingness to learn AI
**Statement 1**	0.432**	0.310**	0.368**	0.162*	0.375**
**Statement 2**	0.326**	0.235**	0.296**	0.202*	0.224*
**Statement 3**	0.293*	0.240*	0.273*	0.130*	0.311**
**Statement 4**	0.284*	0.248*	0.279*	0.190*	0.349**
**Statement 5**	0.279*	0.237*	0.268*	0.181*	0.313**
**Statement 6**	**0.491** **	**0.434** **	0.406**	0.242*	**0.430** **
**Statement 7**	**0.463** **	0.414**	0.361**	0.265**	**0.435** **
**Statement 8**	0.422**	0.373**	0.385**	0.264**	**0.490** **
**Statement 9**	0.406**	0.335**	0.404**	0.260**	0.367**
**Statement 10**	0.423**	0.355**	0.415**	0.264**	0.399**

** = p < 0.001. Statement details can be referred from Table 2.

Another question of whether all students should receive AI training was also found significantly correlated with the positive impact of AI systems in pharmacy education (r = 0.463, p < 0.001) and the willingness of students to learn AI (r = 0.435, p < 0.001). The question of students being confident to use AI after pharmacy education was found significantly correlated to the willingness of students to learn AI (r = 0.490, p < 0.001).

Students at private sector universities stood out in computer literacy compared to public sector universities (χ^2^ = 6.546, p < 0.05). Also, there was a better understanding observed in the students of private sector universities about basic knowledge of AI principles (χ^2^ = 8.716, p < 0.05). All the other responses reported the same understanding, perceptions and willingness to learn AI among the participants of both sector universities (Tables 5 & 6).

**Table 5:** Cross-tabulation of Perception variables with university type.

**Variable**	**Public sector university**							**Private sector university**								**Χ** ^**2**^
Computer literacy level	**Literate**			**Competent**		**Proficient**		**Literate**			**Competent**		**Proficient**			
	110			95		27		124			166		48			**6.546** *
Use of Computer for Learning	**Always**			**Sometimes**		**Never**		**Always**			**Sometimes**		**Never**			
	151			79		2		237			100		1			2.223
**Variable**		**Public sector university**							**Private sector university**						**Χ** ^**2**^	
		**“Strongly agree”**	**“Agree”**		**“Disagree”**		**“Strongly disagree”**		**“Strongly agree”**	**“Agree”**		**“Disagree”**		**“Strongly disagree”**		
**Statement 1**		87	133		11		1		115	201		19		3	1.188	
**Statement 2**		41	142		44		5		81	189		63		5	3.57	
**Statement 3**		26	168		35		3		62	239		36		1	**8.716** *	
**Statement 4**		28	161		40		3		58	228		48		4	3.276	
**Statement 5**		31	152		46		3		57	231		49		1	5.551	
**Statement 6**		65	153		12		2		112	202		24		0	5.729	
**Statement 7**		85	130		14		3		124	183		27		4	0.833	
**Statement 8**		39	149		42		2		78	209		49		2	4.021	
**Statement 9**		35	156		37		4		69	213		52		4	2.834	
**Statement 10**		36	161		29		6		69	213		53		3	6.125	

* = p < 0.05. Statement details can be referred from Table 2.

**Table 6:** Impact of AI on pharmacy education and willingness to use AI compared with type of university.

Variable	Public sector university				Private sector university				Χ^2^
	“Strongly agree”	“Agree”	“Disagree”	“Strongly disagree”	“Strongly agree”	“Agree”	“Disagree”	“Strongly disagree”	
**Statement 1**	67	150	15	0	93	219	26	0	0.380
**Statement 2**	76	149	7	0	110	207	19	2	3.616
**Statement 3**	51	140	41	0	68	189	74	7	6.716
**Statement 4**	42	82	89	10	62	138	122	16	2.477
	**“Strongly willing”**	**“Willing”**	**“Not willing”**	**“Strongly not willing”**	**“Strongly willing”**	**“Willing”**	**“Not willing”**	**“Strongly not willing”**	
**Willingness**	56	157	18	1	95	217	23	3	1.653

** = p < 0.001. Statement details can be referred from Table 3.

## Discussion

The findings of our study revealed prospective pharmacists’ insights toward the incorporation of AI in academic and healthcare settings. The literature review highlighted that no such research has been carried out in Pakistan to evaluate the pharmacy students’ perspectives focusing on an AI-infused future. However, many studies have discovered the opinions of university students on integrating AI into other fields [22,23]. Regardless of the expanding use of AI in pharmaceutical research, there is a paucity of information about AI learning and application in research and practice. This may be related to a slow acceptance rate in pharmacy education or a lack of reporting by pharmacy academicians utilizing AI. There is no denying that students must understand the significance of AI in health education to cope with contemporary pharmacy practice [24].

The current research reported a preponderance of female and younger students. Similar demographic characteristics were described in the study by Buabbas et al., in which the sample’s mean age was 22.1 ± 1.8 years and gender distribution was 39 (11.1%) to 313 (88.9%) [21]. They also reported that most students utilized computer technology in their education and possessed proficient computer abilities. In contrast, our study revealed that students stated to have lower levels of computer literacy and proficiency. The majority of respondents in the present study showed a receptive approach toward using AI in the pharmacy field and indicated a keen interest in AI-infused education. Anderson et al., reported that approximately half of pharmacy students were using AI tools for clinical, academic, or personal purposes. Besides, a large number of students expressed a desire to learn how to use AI tools to support their academic endeavors and believed that AI tools education should be included in their curriculum [25].

AI could revolutionize learning settings in numerous ways, such as intelligent tutoring platforms that identify and fill in knowledge gaps in students, virtual trainers, mining data, and smart feedback [22]. It was evident that our students thought AI applications had the potential to transform pharmacy education and believed that AI implementation would facilitate learning. More than 90% of the respondents in our study admitted that employing AI tools is essential for providing better patient care. Another study reported that more than 80% of the respondents believed that AI assists healthcare professionals in making correct decisions [26]. Parallel to this, Sit et al. stated that 88% of medical students deemed that AI would have a significant impact on the future of medicine [18]. They did, however, indicate in their findings that there was a significant statistical difference in the observed readiness for the application of AI among students who had received prior AI education. It was not surprising that the vast majority of the students in our study anticipated that AI would occupy an important place in the prospects of healthcare. A systematic review depicted that 76% of healthcare students had an affirmative attitude toward AI and its use in the clinical profession future. On the other hand, 24% of the students thought AI posed a threat to the healthcare industry and had a negative attitude toward it [27]. These results are consistent with a larger trend in healthcare, where AI is now viewed as a useful tool to aid in patient care and clinical decision-making. More than 80% of the students in the current study were cognizant of the limitations imposed by AI. Beyond their clinical duties, pharmacists also have leadership and health advocacy responsibilities. To ensure patient welfare, future pharmacists must be ready to confront the substantial ethical and practical issues raised by the disruptive potential of AI in healthcare [19].

It was notable that more than 90% of our respondents intend to get AI training while pursuing a pharmacy degree and 83% of the students consider that knowledge of AI will assist them in the accomplishment of their professional goals. This is consistent with outcomes from studies investigating students’ viewpoints towards practicing AI in healthcare, where 69–90% of participants expressed favorable opinions, pointing to a similar tone of optimism about the significant opportunities of AI in different healthcare domains [28–30]. Consequently, pharmacy curriculum developers must take the initiative to get students ready for cutting-edge procedures in drug design and drug delivery. Previous studies have revealed that students who had received prior education or training showed a stronger willingness to apply AI in clinical settings and have an advanced level of confidence in their knowledge of the concepts underlying AI [17,31]. Getting prior training in AI is associated with a higher level of credence in their capability to practice AI tools in their fields. A study reported that students who were taught about digital health throughout their pharmacy school years were 2.5 times more likely to say they had received ongoing instruction in the field [32]. This implies that early exposure to digital health issues may play a significant role in fostering a desire for further education later.

The future of pharmacy strongly relies on the application of AI technology, which will strive towards the goal of ensuring that everyone has access to good health and well-being. However, it has been revealed that a significant number of pharmacy schools do not provide AI education [9]. There is a knowledge and skill gap in the application of AI technology to address current clinical issues and enhance patient care. Our findings indicated a higher inclination of students in AI; however, the majority of the pharmacy students reported that they weren’t involved in any AI-related curricular activities. These results are similar to a Saudi study presented by Syed *et al*., in which 80% of pharmacy students revealed that no formal AI training had been provided [26]. Hence, there should be avenues to provide more knowledge about these technological advances for all prospective pharmacists. This could be accomplished through introductory simulation-based learning to students as mandatory or optional courses addressing themes including big data analytics, health-related uses of AI, ethical considerations, ethical constraints, and implications for the future. Collaboration across pharmacy schools could be a strategy to accelerate the adoption of AI health education and reduce variations in the preparedness and responsiveness of pharmacy education and training.

A UK study reported that medical students expressed their familiarity with the fundamental computational concepts of AI and its limits; however, they felt uncomfortable applying the AI vocabulary [18]. Another study conducted on pharmacy faculty and students reported that participants comprehended AI nomenclature better than AI benefits, suggesting that individuals may know more phrases and concepts related to AI than its possible advantages and disadvantages. This discrepancy may result from a lack of formal education and training in AI [24]. By contrast, our study reported that 83% of respondents were confident using AI tools. In the current study, only 17% of students negated that they understand pharmacy concepts better with the help of AI. Another study reported that nearly 50% of pharmacy students expressed concerns about the increasing ethical and legal challenges surrounding the application of AI in medicine and felt inadequate to deal with AI in their imminent careers [19].

Pharmacists and patients will not be able to fully enjoy the benefits of digital health until they have a firm grasp of the principles behind AI technologies and know how to incorporate them into their clinical practices. A competency-based curriculum for digital health should be designed to give students the information and abilities they need to practice digital health in the workplace [8]. In the current study, 93% of the students assured that the assimilation of AI into the curriculum will aid in improving student comprehension and prepping them for real-world clinical settings. It is a need for hours that the next generation of pharmacists must be well aware of the advanced clinical approaches and their execution. AI applications require innovative capabilities for exploring and predicting outcomes in drug discovery. In the current study, there were students’ varying opinions about AI taking over pharmacist duties in the future. Another study reported that 23% of respondents thought that AI would curtail the humanistic aspects of the medicinal profession [26]. Jha et al., also found that 24.1% of physicians and medical students differed that AI will decrease the workforce [33]. Pharmacy education must foster students about the prospects of more growth and innovative roles through the impending integration of AI into clinical practice. AI systems might be designed, for instance, to analyze and evaluate medication regimens for potential risks to patients [34]. A global survey revealed that all pharmacy students should graduate with a foundational understanding of patient-centered digital health. Specialization and ongoing professional development are essential to upskilling and training the current pharmacy workforce in digital health [32].

The Accreditation Council for Pharmacy Education has not yet covered AI competencies within its standards for pharmacy schools. Nonetheless, a report published in Academic Medicine provided certain recommendations for the competencies of medical professionals—including pharmacists—who will employ AI-based tools in their practice [9]. Our findings indicated a positive correlation between attitudes about AI and knowledge of AI; indicating that knowledge is a crucial factor in influencing behavior change toward the use of AI in pharmacy practice. Furthermore, we found a strong positive association between student’s perception of AI and their willingness to use it. This suggests that raising students’ understanding and familiarity with AI could result in a greater willingness to embrace and use AI tools in pharmacy practice. This research would serve as a platform for highlighting the need to assimilate AI in pharmacy learning and act as a foundation for the desired future investigations. Educational and healthcare organizations may potentially utilize these outcomes for developing AI training programs.

The outcomes of the current study might have been predisposed towards certain limitations. Firstly, only final-year pharmacy students participated in the study. Hence, the findings could not be extrapolated to other student populations at this university or any other in Pakistan. Secondly, the study was conducted as a self-administered online survey, we depended on respondents to truly record their responses which could have led to bias. Furthermore, the cross-sectional study’s design has other limitations, such as the incapacity to evaluate causal correlations. Notwithstanding these limitations, the study offers insightful information about students’ viewpoints. Subsequently, we contemplate that the outcomes are valuable to provide direction to health and education policymakers.

## Conclusion

The findings of the present study revealed the higher willingness of pharmacy students towards AI-infused learning. They understood the prerequisite of having both formal and informal learning experiences on the clinical application, technological constraints, and ethical considerations of the AI tools to be successful in this endeavor. Furthermore, most students acknowledged that AI might positively impact pharmacists’ role and improve the efficiency of the healthcare system. Given the relevance and significant implications of AI, policymakers must take action to ensure that future pharmacists have a strong foundation of AI literacy and take initiatives to foster the interests and abilities of imminent pharmacists who will spearhead innovation in the field.
